# Safety of switching between rituximab biosimilars in onco-hematology

**DOI:** 10.1038/s41598-021-85563-1

**Published:** 2021-03-16

**Authors:** Silvana A. M. Urru, Stefania Spila Alegiani, Anna Guella, Giuseppe Traversa, Annalisa Campomori

**Affiliations:** 1 Hospital Pharmacy Unit, Trento General Hospital, Autonomous Province of Trento, Trento, Italy; 2 School of Hospital Pharmacy, University of Sassari, Sassari, Italy; 3 Pharmacoepidemiology Unit, National Center for Drug Research and Evaluation, Italian National Institute of Health, Rome, Italy; 4 Hematology Unit, Trento General Hospital, Autonomous Province of Trento, Trento, Italy

**Keywords:** Cancer, Medical research, Lymphoma, Antibody therapy

## Abstract

Comparable clinical efficacy and safety of the reference rituximab (MABTHERA) and its biosimilars has been established in randomized trials. However, safety concerns are often raised when switching from reference to biosimilar products and between different biosimilars. In this prospective observational study we aimed at evaluating the safety of switching between reference and biosimilar rituximab (TRUXIMA and RIXATHON) at Trento General Hospital (Italy). All patients (n = 83) with Non Hodgkin’s Lymphoma (NHL, n = 72) and Chronic Lymphocytic Leukemia (CLL, n = 11) who received rituximab between March 2018 and March 2019 were asked to take part in the study. In 2017 and 2018 two tenders were carried out and two different biosimilars became available in the hospital, these were used sequentially. Thus, patients with or without previous treatments with the originator rituximab either received a biosimilar or were switched between different biosimilars. The incidence of adverse events in these groups of patients is described. The study population received 465 rituximab infusions and all received biosimilars. Fifty patients (60%) experienced at least one switch between different biosimilars or between rituximab originator and biosimilar, whereas 33 (40%) received one of the two biosimilars and one patient received reference rituximab. Adverse events (n = 146) were reported in 71 patients (84.5%). Treatment-related grade 3–4 events were reported in 5 patients (5.9%), whereas grade 1 rituximab related infusion events were observed in 6 patients (7.1%). No safety signal emerged in association with the use of a specific biosimilar nor with the practice of switching. Adverse events were similar, in terms of seriousness and frequency, to those described in the literature, providing further support to the clinical safety of rituximab biosimilars.

## Introduction

Biosimilars are approved on the basis of a comprehensive comparability exercise aimed at establishing the similarity to the reference medicinal products in terms of quality, biological activity, safety and efficacy^1^.

The first biosimilars introduced in Europe in 2006, were biosimilar somatropins^2^. Until recently, only biosimilars of these lower molecular-weight biologics were available. This changed in September 2013 when the European Medicines Agency (EMA) recommended the granting of marketing authorization for the first time for two biosimilar versions of the monoclonal antibody (mAb) infliximab^3^.

As of 1st January 2021, there are 58 authorized products in the EU and 29 in the USA^4^. Existing data on switching for selected biosimilars have been generated from several studies^5–7^. In more than 10 years of clinical experience, no substantial clinical and safety differences have been detected^8^. However, especially for newly marketed biosimilars, concerns are raised with respect to the practice of switching in patients already treated with a specific biologic product (either reference or biosimilar)^9^.

Differently for the FDA, the EMA does not make a distinction between biosimilars and interchangeable products and advice to prescribers fall under the responsibility of member states^10^.

In Europe, biosimilars have become a reality, with some biosimilars achieving market share of > 90%, while in the USA, the uptake of biosimilars has been modest thus far^11^.

The first biosimilar rituximab was approved in 2017^9^. The equivalence between reference rituximab (MABTHERA) and its biosimilars—in terms of pharmacokinetics, pharmacodynamics, efficacy, safety and immunogenicity—has been demonstrated in randomized, double-blind, controlled trials^12–15^.

After a careful review of the scientific evidence on rituximab, hematologists and pharmacists working at Trento General Hospital agreed that reference and biosimilar products could be used interchangeably in all patients, both naïve and experienced ones. Consequently, in 2017 and 2018 two tenders were carried out and two different biosimilars became available in the hospital: TRUXIMA (Mundipharma) in the first year and RIXATHON (Sandoz) in the second one. It was also agreed to conduct a prospective observational study specifically focusing on safety to build patient and physician confidence.

The aim of the study was to document any adverse event (AE) reported in association with the use of biosimilar rituximab and with the practice of switching between different products in patients with Non Hodgkin’s Lymphoma (NHL) and Chronic Lymphocytic Leukemia (CLL).

## Methods

The study was conducted in accordance with ethical principles derived from guidelines that included the Declaration of Helsinki^16^, as well as following all relevant local requirements. The Ethics Committee of the Health Trust of the Autonomous Province of Trento approved the study protocol (2018/n.4586). Informed consent was secured from all subjects in this study. Patients were treated according to the usual practice and no additional procedure was carried out. Each patient was informed about the objectives of the study and provided written informed consent to collect and analyze data for research purposes.

The study population consisted of adult patients with NHL and CLL, consecutively admitted into the Hematology Unit of the Trento General Hospital from March 10th 2018 to March 10th 2019, whose therapy included rituximab administration. Patients were followed up until the first of the following dates: last follow up visit, death, end of study (30 September 2019).

Baseline characteristics were ascertained at the time of the first infusion of rituximab (dosed at 375 mg/m^2^ as part of standard treatment), during the study period. Data were collected on patient characteristics (i.e., diagnosis, age, body surface area, performance status), previous treatment and concomitant medications, premedications, rituximab indications, dosage and administration.

During the study period, patients may have received: (a) either a biosimilar or the reference product (no-switch group); (b) two biosimilar products (switch during the study period); (c) a rituximab formulation (reference or biosimilar) that was different from the one received before the study period (anamnestic switch).

Adverse events (AEs) of interest consisted of Infusion Related Reactions (IRR) (i.e., type of reaction, treatment of reaction, duration of interruption of infusion) and other adverse events occurring between different infusions, regardless of their severity (grade 1–4). Safety follow-up took place at every administration of rituximab; information on adverse events occurring at home was obtained at every clinical access (at least once per month). The causality assessment for all drug–event couples was made by the attending physicians using the Naranjo algorithm^17^.

NHL and CLL disease activity was assessed according to the local clinical practice routine, after the third cycle (week 9) and at the end-of-treatment visit, and was grouped as overall response rate, complete response, partial response, stable disease, progressive disease^18,19^.

Performance status was assessed by clinicians using the Eastern Cooperative Oncologic Group Scale (ECOG) scale^20^.

We described the characteristics of patients included in the study using counts with percentages and median with interquartile range (IQR) for categorical and continuous variables, respectively. The incidence of AEs among patients with “no-switch”, “switch during the study period” and “anamnestic switch” was analysed through a Chi-square test for categorical variables. Both the number of patients and the number of infusions were used as denominator of the events of interest. The study population represented the experience of a single hospital and no formal sample size calculation was carried out.

## Results and discussion

Eighty-three patients (37 women and 46 men) affected by NHL (n = 72) and CLL (n = 11) were included in the study (Table 1). Patients had a median age of 71 years (interquartile range-IQR 63–79 years) and more than 20% had a performance status ≥ 3. The median follow-up of the patients was 10.5 months (IQR 7–14 months).

**Table 1:** Characteristics of patients.

N. of patients	83
Female [n (%)]	37 (44.0%)
BSA (m^2^) [median (IQR)]	1.8 (1.7–1.9)
Age at diagnosis (years) [median (IQR)]	68 (62–78)
Age at baseline (years) [median (IQR)]	71 (63–79)
**Diagnosis** **[n (%)]**	
NHL	72 (86.9%)
Indolent NHL	
Follicular lymphoma	19 (23.8%)
Extranodal marginal zone (MALT)	1 (1.2%)
Aggressive NHL	
Diffuse large B-cell CD20 positive	51 (60.7%)
Mantle cell lymphoma	1 (1.2%)
CLL	11 (13.1%)
**Performance status** **[n (%)]**	
0	4 (4.8%)
1	27 (32.1%)
2	33 (39.9%)
≥ 3	17 (20.2%)
NA	2 (3.6%)
**Cycles of rituximab, total cycles n. 465** [n (%)]	
Biosimilar-TRUXIMA	163 (34.9%)
Biosimilar-RIXATHON	302 (63.6%)
**Switch, total patients n. 83** **[n (%)]**	
No switch	33 (40.5%)
Switch during the study period	26 (31.9%)
Anamnestic switch	24 (28.6%)
**Response, NHL [n (%)]**	
Complete response	60 (83.6%)
Partial response	7 (9.6%)
Progressive disease	3 (4.1%)
Not evaluated (ongoing treatment)	2 (2.7%)
**Response, CLL** **[n (%)]**	
Complete response	2 (18.2%)
Partial response	2 (18.2%)
Progressive disease	2 (18.2%)
Stable disease	2 (18.2%)
Not evaluated (ongoing treatment)	3 (27.2%)

*n* number, *BSA* body surface area, *IQR* inter quartile range, *NHL* Non Hodgkin’s Lymphoma, *CLL* chronic lymphocytic leukemia, *NA* not available.

During the study period the patient population received 465 infusions of intravenous rituximab (163 TRUXIMA, and 302 RIXATHON). The median dosage received was 652 mg (range 500–900 mg). The median number of infusions per patient was 5.6 (range 1–8 infusions). All patients (n = 83) received biosimilars. Among non-switchers, 33 patients (40%) received a biosimilar formulation. At least one switch was experienced by the remaining 50 patients (60%): 26 (31%) during the study period and 24 (29%) before the study period (anamnestic switch).

Adverse events (n = 146) were reported in 71 patients (85.5%). Fifty-five (66.3%) and 10 (12.0%) patients had respectively neutropenia or anemia of grade 1–2. Treatment-related grade 3–4 AEs were reported in five patients (6.0%): neutropenia in two patients, and febrile neutropenia, thrombocytopenia, liver toxicity, in one patient each. Six patients experienced rituximab related adverse events of grade 1, which is consistent with the scientific literature (Table 2)^21^.

**Table 2:** Hematologic and non-hematologic adverse events registered during the study period^a^ (March 2018- March 2019).

Adverse events	Any grade	Grade 3–4	Rituximab related AE^b^ Grade 1–2
	n (%)	n (%)	n (%)
**Hematologic**			
Neutropenia	57 (68.7)	2 (2.4)	–
Anemia	10 (12.0)	–	–
Febrile Neutropenia	2 (2.4)	1 (1.2)	–
Thrombocytopenia	2 (2.4)	1 (1.2)	–
**Non hematologic**			
Fever	13 (15.7)	–	2 (2.4)
Tingling of the hands or feet	7 (8.4)	–	–
Rash	6 (7.2)	–	–
Urinary tract infection	5 (6.0)	–	–
Nause	4 (4.8)	–	–
Constipation	4 (4.8)	–	–
Cough	2 (2.4)	–	–
Stuffy nose	2 (2.4)	–	2 (2.4)
Liver toxicity	1 (1.2)	1 (1.2)	–
Throat itching	1 (1.2)	–	1 (1.2)
Back pain	1 (1.2)	–	1 (1.2)
Dyspnea	1 (1.2)	–	1 (1.2)
Tremors	1 (1.2)	–	1 (1.2)
Headache	1 (1.2)	–	1 (1.2)
Other	26 (31.3)	–	–

*N* number.

^a^Events experienced by at least two patients, or grade 3–4, or causally related to rituximab, are reported in the table.

^b^The events were assessed as causally related to rituximab by the attending clinicians.

The incidence of AEs was similar in patients who received one or two biosimilar formulations, both for any events (32/33 patients in the no switch group vs 25/26 patients with a switch during the study period, p = 0.86) and for events of grade 3–4 (2/33 vs 1/26; p = 0.70).

The proportion of AEs was lower in patients who were receiving a rituximab formulation (one biosimilar or the other) that was different from the one before the study period (14 out of 24 patients, 58%; 2 events of grade 3–4) (data not shown).

After a median follow-up of 10 months, adverse events reported were similar in terms of seriousness and frequency, regardless of rituximab formulation and switching. The incidence of events was lower only in the group of prevalent patients who had been already treated in the past with a different rituximab formulation (anamnestic switching).

To our knowledge, this is the first real‐life cohort study assessing the safety of switching between different rituximab formulations (biosimilars and originator) in NHL and CLL patients.

Although some open-label studies have shown an increased number of withdrawals or AEs following a switch, these outcomes were less frequently observed in randomized studies^9,21^ suggesting the potential occurrence of a “nocebo” effect resulting from negative expectations toward the biosimilar^22^.

The results of this study support the position that switching between biosimilars, or from reference rituximab to its biosimilars, as part of routine clinical practice in NHL and CLL patients, has the same safety profile expected in patients continuously treated with reference rituximab. Data from post-marketing studies and real-world experience are needed to provide additional information to supplement the strong evidence already obtained on biosimilars from RCTs.

The increasing availability of biosimilars has led to significant healthcare savings and provided greater patient access to high cost therapeutics^23^. However, the cost-saving potential depends on various factors, such as the price of the reference product and the competition market^24^.

A cost-analysis study conducted in Europe, predicted that switching to a rituximab biosimilar would save €56.82 million over a year^25^.

In the setting of our hematology unit of a general hospital, this shared approach has increased clinicians and patients confidence in biosimilars with respect to safety, generating at the same time a 45% reduction in the price of rituximab (around 400,000 euros savings in one year).

## Data Availability

The data that support the findings of this study are available on request from the corresponding author [S.U.]. The data are not publicly available because they contain information that could compromise research participant privacy/consent.
